# Irregular screening participation increases advanced stage breast cancer at diagnosis: A population-based study

**DOI:** 10.1016/j.breast.2022.07.004

**Published:** 2022-07-08

**Authors:** L. Ding, M.J.W. Greuter, I. Truyen, M. Goossens, H. De Schutter, G.H. de Bock, G. Van Hal

**Affiliations:** a Department of Epidemiology, University of Groningen, University Medical Center Groningen, Groningen, the Netherlands; b Department of Social Epidemiology and Health Policy, University of Antwerp, Antwerp, Belgium; c Department of Radiology, University of Groningen, University Medical Center Groningen, Groningen, the Netherlands; d Department of Robotics and Mechatronics, University of Twente, Enschede, the Netherlands; e Belgian Cancer Registry, Brussels, Belgium; f Center for Cancer Detection (CvKO) in Flanders, Belgium; g Vrije Universiteit Brussel, Brussels, Belgium

**Keywords:** Breast neoplasms, Digital mammography, Neoplasm staging, Screening participation, Regularity

## Abstract

**Objective:**

To evaluate the effect of irregular screening behaviour on the risk of advanced stage breast cancer at diagnosis in Flanders.

**Methods:**

All women aged 50–69 who were invited to the organized breast cancer screening and diagnosed with breast cancer before age 72 from 2001 to 2018 were included. All prevalent screen and interval cancers within 2 years of a prevalent screen were excluded. Screening behaviour was categorized based on the number of invitations and performed screenings. Four groups were defined: regular, irregular, only-once, and never attenders. Advanced stage cancer was defined as a stage III + breast cancer. The association between screening regularity and breast cancer stage at diagnosis was evaluated in multivariable logistic regression models, taking age of diagnosis and socio-economic status into account.

**Results:**

In total 13.5% of the 38,005 breast cancer cases were diagnosed at the advanced stage. Compared to the regular attenders, the risk of advanced stage breast cancer for the irregular attenders, women who participated only-once, and never attenders was significantly higher with OR_adjusted_:1.17 (95%CI:1.06–1.29) and OR_adjusted_:2.18 (95%CI:1.94–2.45), and OR_adjusted_:5.95 (95%CI:5.33–6.65), respectively.

**Conclusions:**

In our study, never attenders were nearly six times more likely to be diagnosed with advanced stage breast cancer than regular attenders, which was much higher than the estimates published thus far. An explanation for this is that the ever screened women is a heterogeneous group regarding the participation profiles which also includes irregular and only-once attenders. The benefit of regular screening should be informed to all women invited for screening.

## Highlights

• Irregular screening significantly increases the risk of advanced stage breast cancer at diagnosis.
• Never attenders were six times more likely to be diagnosed with advanced stage breast cancer than regular attenders.
• Women should be encouraged to participate in screening regularly.

## 1 Introduction

Female breast cancer is a commonly diagnosed cancer, representing one fourth of all newly diagnosed cancers in women worldwide [1]. The stage of breast cancer at diagnosis is a significant prognostic factor for the overall survival rate for breast cancer [2]. The five-year survival rate for stage I breast cancer has approached 100%, but declines to less than 30% for stage IV breast cancer [2,3]. A contributing factor for the observed decrease in breast cancer mortality is the shift to early stages of breast cancer at diagnosis [4]. At the population level, earlier stage diagnosis of breast cancer can be achieved by implementing a breast cancer screening programme, with sufficient quality and participation rates [5,6]. In many European countries, mammography screening is offered in a systematic way in population based programmes, but co-exists alongside opportunistic screening [7], in which mammograms are offered at women's request or during regular healthcare checkups [6].

In randomized controlled trials (RCTs), the non-screened control groups have a higher risk of advanced stage breast cancer than the screened group [8,9]. The effect of screening on cancer stages at diagnosis at the population level has been evaluated in some ecological studies, in which a reduction of advanced stage breast cancer incidence has been observed in women who participated in screening compared with non-participants [5,10]. Some studies that used data at an individual level have also indicated that non-participation is associated with advanced breast cancer stages [[11], [12], [13]]. However, in these studies the breast cancer stages at diagnosis were only compared between the ever and never screened women [[11], [12], [13]]. Within the ever screened group, women could have participated in screening with variable intervals between consecutive rounds, impacting screening regularity. However, such detailed investigation of screening regularity requires the linkage of data of the invited women at individual level from multiple sources, which can be difficult to perform. In published studies thus far, no quantitative evidence is available about the effect size of regular screening on the risk of advanced stage breast cancer at diagnosis.

The aim of this study was, therefore, to evaluate the association between stage at time of diagnosis and breast cancer screening regularity, using individual level data from women eligible for breast cancer screening residing in Flanders, Belgium.

## 2 Method

### 2.1 Breast cancer screening in flanders

Since 2001, the population-based organized breast cancer screening programme has been implemented by the Center for Cancer Detection (CCD) in the whole region of Flanders. All women aged 50–69 with no history of breast cancer are eligible to participate biennially. The cost of the organized screening is fully covered by the universal health insurance system in Flanders. The quality of the organized screening programme is ensured by systematic quality control measures, following European guidelines [14]. Besides the organized screening programme, opportunistic screening may be performed on the spontaneous initiative of the woman or her physician. The opportunistic screening practices existed even before the organized screening programmes and have remained an option for screening for a large proportion of women ever since. In 2016, the percentage of eligible women who were covered by the organized and opportunistic screening was 50.0% and 14.1%, respectively [15]. Of note, opportunistic screening is not subject to systematic quality control, and is only partially reimbursed by the health insurance system. The organized screening programme in Flanders invited all eligible women until the year 2017.

Since opportunistic screening covers a sizeable proportion of women who are eligible for screening, the Belgian Cancer Registry (BCR) includes both the organized and opportunistic screening in participation profiles. An opportunistic screening mammogram was defined as a mammogram performed outside the organized screening programme. However, mammograms that occurred within 3 months following a positive organized screening and/or within 3 months prior to cancer diagnosis were recognized as the diagnostic mammograms for the confirmation of breast cancer diagnosis rather than the opportunistic screening mammogram. All mammograms performed after a breast cancer diagnosis are not relevant to screening and were not taken into consideration.

### 2.2 Study design and data sources

The study cohort was constructed using individual level data from the CCD in Flanders, the BCR, and the InterMutualistic Agency (IMA). All data were routinely collected within the context of the organization and evaluation of the organized breast cancer screening programmes, as defined in the legal tasks of each data provider involved [16]. The CCD in Flanders provided the data on the participation in the organized screening programme from 2001 to 2017. The IMA collects all data of reimbursement health care from the universal health care system [17]. Whenever women participated in opportunistic mammography screening, the payment will be partially reimbursed by health insurance and the data will be transferred to the IMA database. For this study, the IMA provided information on mammograms outside the organized screening programme from 2001 to 2017. In addition, IMA data indicated persons who could benefit from increased reimbursement at the first invitation to screening of each woman, serving as a proxy for a weaker socio-economic position. Since an increased reimbursement is the social aid granted by the social security system for people who have experienced economic hardship, for women who have an increased reimbursement, a low socioeconomic status (SES) can be expected. The cancer diagnoses data were provided by the BCR and covered diagnoses in Flemish residents for the years 2001–2018. All women were informed that they could freely choose to refuse their data being used for research at the time of screening. The percentage of screened women who opt-out their data from research fluctuates around 1% [15]. All data were deterministically linked, using the national social security number as a unique personal identifier, according to existing data flows that are exerted in line with general data protection regulations (GDPR). Only pseudonymized data were used for this study, and results are reported in an aggregated way.

### 2.3 Definition of population, outcome and determinants

#### 2.3.1 Population

The population for this study consisted of all women who were invited for organized breast cancer screening in Flanders and diagnosed with breast cancer from 2001 to 2018. Since only the information of breast cancer diagnosis between 2001 and 2018 was available, we only included women who had their last screening between 2001 and 2016 to ensure all women have a maximum 24 months of follow-up time after the last screening and identify breast cancers related to screening. Since women older than 69 were no longer invited to screening, we only included women who were diagnosed with breast cancer before age 72. Moreover, we excluded women who were only invited once, since the regularity of screening cannot be determined with a single screening invitation. All prevalent screen and interval cancers within 2 years of a prevalent screen were therefore excluded (Table S1).

#### 2.3.2 Outcome

The outcome was the breast cancer stage at diagnosis, for breast cancers diagnosed prior to the age of 72. If multiple lesions were found in a woman, we only retained the most advanced lesion for the analyses (e.g. prioritising the invasive over the in situ lesion). A combined stage was considered in which pathological stage prevails over clinical stage, except for distant metastases, which were always considered stage IV. Stage was defined according to the applicable TNM edition [18,19]. Stages of breast cancer were determined at diagnosis before any treatment. A minor number of breast cancers were only recorded after neoadjuvant therapy and had reduced stage. As the stage at diagnosis for these breast cancers were not known, they were classified as stage unknown in the database by the BCR. We considered stages III and IV as advanced stages and stage I, II, and carcinoma in situ as early stages. For breast cancers with unknown stages, the distribution of participation profiles of these cases was demonstrated in the descriptive analyses but not included in the regression models.

#### 2.3.3 Determinants

The main determinant was the screening profile. A woman was considered *a regular attender* if she attended the organized and/or opportunistic screening at least twice, and the uptake was ≥70%, and the average interval of the attended mammography screening was between 20 and 28 months. The uptake of screening was used to ensure each woman had sufficient number of screenings, based on the similar idea of the 70% acceptable level of participation rate at the population level recommended by the EU guidelines [14], and defined as the number of screenings attended, divided by the total number of screening opportunities. The total number of screening opportunities was determined by the length of time each woman was eligible for screening and the biennial screening interval. For women who were diagnosed with breast cancer before age 69, the endpoint of the eligible period was the time at breast cancer diagnosis. The average interval of the attended mammography screenings was defined as the length between the first and the last screening divided by the number of screenings. The average interval was defined as 20–28 months, rather than the fixed 24 months, in order to depict the flexibility of screening which could be rescheduled on women's demand. A woman was considered *an irregular attender* if she attended the organized and/or opportunistic screening at least twice, and the uptake was less than 70%, and/or the average interval was less than 20 months or over 28 months. A woman was considered as a *once in screening* when she participated only once in the screening after at least two invitations. All other women who never performed a screening after at least two invitations was categorized as *never attenders.* The multivariable logistic regression models were adjusted for age at diagnosis, and the SES, where an increased reimbursement status was used as an indicator for low SES.

### 2.4 Statistical analysis

The included women were stratified by their age at diagnosis, their screening participation profile, their breast cancer stage at diagnosis, and their increased reimbursement status. Data were reported as numbers and percentages. The association between the screening participation profiles and the risk of advanced stage breast cancer at diagnosis was first evaluated in a univariate logistic regression model, and consequently in a multivariable logistic model with adjustment for age at diagnosis and SES, which in literature has proven to be related to participation in screening and the stage of breast cancer at diagnosis [20,21]. The regularly screened women were used as the reference group in the regression model. The odds ratio (OR) and 95% confidence interval (CI) were reported for the risk of breast cancer diagnosed at the advanced stage.

Since the study population only included women with a diagnosis of breast cancer, overdiagnosis may lead to the diagnosis of more early stage breast cancers in the included breast cancers. Hence the relationship between participation regularity and the risk of advanced stage breast cancer at diagnosis can be biased. To evaluate the effect of overdiagnosis on the association between screening regularity and the risk of advanced stage breast cancer at diagnosis in our estimation, a sensitive analysis assumes a 10% overdiagnosis rate derived from the Dutch population [22,23] was performed, since the level of overdiagnosis in Flanders breast cancer screening programme has not been reported in literature, we applied the data from the Dutch population which is geographically nearby the Flanders region [[24], [25], [26], [27], [28], [29], [30], [31]]. In this sensitivity analysis, a random 10% of early stage screen-detected breast cancers were excluded and the modeling was done in the rest of the cases, since by definition, overdiagnosis is due to the detection of breast cancer that are not progressive at early stage by screening mammograms. To evaluate the robustness of the effect of screening regularity on the risk of advanced stage breast cancer at diagnosis, an additional sensitivity analysis was performed in which advanced breast cancer defined as stage II or above. All statistical tests were two-sided with a significance level at 0.05. All analysis was performed in R 4.0.5.

## 3 Results

In total 38,005 women were diagnosed with breast cancer before age 72 from 2001 to 2018. The average follow-up years ranged between 6.4 years and 11.9 years for never attenders after at least two invitations and for women who were regularly screened, respectively. Of the diagnosed women, the total percentage of advanced breast cancer was 13.5%. Only 9.1% of breast cancers were diagnosed at advanced stage in the regularly screened women, which was lower than the 9.8% of the advanced stage breast cancer in the irregularly screened women. For women who only participated once in screening after at least two invitations, 16.3% of breast cancer were diagnosed at the advanced stage. Never attenders after at least two invitations had more than 30% of advanced stage breast cancer at diagnosis (Table 1). More advanced stage breast cancers were diagnosed in old women than the young ones (Table 1).

**Table 1:** The number of women diagnosed with breast cancers and the percentage of breast cancers diagnosed at advanced stage in women of different participation profiles, incidence age groups, and increased reimbursement status.

Variable	BC cases	Advanced cases	
	Num	Num	%^a^
**Total**	38,005	5149	13.5
**Age group of women at breast cancer diagnosis**			
50-54	4221	457	10.8
55-59	9957	1295	13.0
60-64	10,595	1492	14.1
65-71	13,232	1905	14.4
**Screening participation**			
**Regular**	5825	532	9.1
**Irregular**	22,019	2156	9.8
**Participated only once**	6018	982	16.3
**Never attended**	4143	1479	35.7
**Increased reimbursement status**^b^			
Yes	4571	726	15.9
no	33,434	4423	13.2

a Row percentages were calculated for women in different groups.

b An increased reimbursement status indicates women who are likely to have a low socioeconomic status.

The multivariable logistic regression model showed that the risk of advanced stage breast cancer for the irregular attenders was higher than in the regular attenders, with OR: 1.17 (95%CI: 1.06–1.29) (Table 2). In the group who only participated once after at least two invitations, the risk of breast cancer diagnosed at an advanced stage was also higher than for regular attenders, with OR: 2.18 (95%CI: 1.94–2.45). Never attenders after at least two invitations had the highest risk of advanced stage breast cancer at diagnosis with OR: 5.95 (95%CI: 5.33–6.65) (Table 2).

**Table 2:** Association between screening regularity and the risk of advanced stage breast cancer at diagnosis.

Participation regularity	Model 1			Model 2		
	OR	95%CI	*P*	OR	95%CI	*P*
**Regularly screened**	ref	–	*-*	ref	–	*-*
**Irregularly screened**	1.10	(1.00–1.22)	0.060	1.17	(1.06–1.29)	<0.001
Participated only once*	2.00	(1.79–2.24)	<0.001	2.18	(1.94–2.45)	<0.001
Never attenders*	5.75	(5.15–6.42)	<0.001	5.95	(5.33–6.65)	<0.001

Model 1: univariate model.

Model 2: multivariable model adjusted with age of women at breast cancer diagnosis, and socioeconomic status.

In the sensitivity analyses, assuming a 10% overdiagnosis rate, the effect size of irregular screening and never attenders decreased slightly to OR: 1.15 (95%CI: 1.04–1.27) and OR: 5.63 (95%CI: 5.04–6.30), respectively (Table 3). The sensitivity analysis with the stage II + breast cancer defined as advanced stage showed that the irregular attenders and never attenders remained statistically significantly more likely to be diagnosed with advanced stage breast cancer than the regular attenders, and the effect size only had a minor change (Table 3).

**Table 3:** The effect of considering overdiagnosis and applying a different definition of advanced stage on the association between screening regularity and the risk of advanced stage breast cancer at diagnosis.

Participation regularity	Model 1			Model 2		
	OR	95%CI	*P*	OR	95%CI	*P*
**10% overdiagnosis rate**						
Regularly screened	ref	–	*-*	ref	–	*-*
Irregularly screened	1.08	(0.98–1.20)	0.120	1.15	(1.04–1.27)	0.010
Participated only once^a^	1.95	(1.75–2.19)	<0.001	2.12	(1.89–2.38)	<0.001
Never attenders^a^	5.45	(4.88–6.08)	<0.001	5.63	(5.04–6.30)	<0.001
**Stage II** + **breast cancer as an advanced stage**						
Regularly screened	ref	–	*-*	ref	–	*-*
Irregularly screened	1.12	(1.06–1.20)	<0.001	1.18	(1.11–1.26)	<0.001
Participated only once^a^	1.98	(1.83–2.13)	<0.001	2.11	(1.95–2.28)	<0.001
Never attenders^a^	5.92	(5.41–6.49)	<0.001	6.07	(5.54–6.65)	<0.001

Model 1: univariate model.

Model 2: multivariable model adjusted with age of women at breast cancer diagnosis, and socioeconomic status.

a For those who received at least two invitations.

## 4 Discussion

### 4.1 Principal findings and comparison with published studies

In this study, we evaluated the effect of breast cancer screening regularity in women aged 50–69 years on the risk of breast cancer diagnosed at advanced stage. Irregular screening increased the risk of advanced stage breast cancer at diagnosis by 17% compared to regular screening. Women who participated only once in screening were twice more likely to be diagnosed with advanced stage breast cancer than the regular attenders. The never attenders had nearly six times higher risk of being diagnosed with advanced breast cancer than the regular attenders. Assuming a 10% overdiagnosis rate, the irregular attenders and never attenders remained statistically significantly related to higher risk of advanced stage breast cancer at diagnosis with the effect size slightly decreased.

In the literature, never attenders have a higher odds ratio of being diagnosed with advanced stage breast cancer than ever screened women, with the reported effect size ranging from 1.41 to 2.05 [21,[32], [33], [34], [35]]. In our analysis, the risk of advanced stage breast cancer at diagnosis was nearly six times higher for the never attenders than the regularly screened women. This may be because we compared the never attenders with the regularly screened women rather than just ever screened women. The finding suggests that previous studies underestimate the effect of regular screening, as they grouped women who only participated once in screening with the regularly and the irregularly screened women. Although overdiagnosis in screening may increase the percentage of early stage breast cancer at diagnosis hence affect the association between screening regularity and the risk of advanced stage breast cancer at diagnosis, we found that never attenders remained more than 5 times more likely to be diagnosed at advanced stage breast cancer than regularly screened women even when 10% overdiagnosis was adjusted in the sensitivity analyses.

### 4.2 Strengths and limitations

The strength of this study is that the participation data and the breast cancer stages were available at the individual level. We applied a strict definition of screening regularity with both the number of screenings and the interval between screenings considered. The regularity of screening was determined based on a longitudinal history of screening, adding granularity to the assessment of the effect on the risk of advanced stage breast cancer at diagnosis, as compared to previous reports purely discriminating ever from never screened women. Moreover, the inclusion of the participation data in the opportunistic screening contributed to a more comprehensive evaluation of the effect of screening.

The study also has some limitations. First, due to privacy regulations, the comparison was made within the women who were diagnosed with breast cancer, not within the population invited for breast cancer screening. Therefore, the effect size measured by odds ratios in our study cannot be interpreted as the probability of advanced breast cancer. Another limitation is that we did not have access to the tumor grade on an individual level. For that we were not able to just assess grade 2 and 3 invasive cancers in the estimated risk of overdiagnosis. However, the 10% overdiagnosis in the sensitivity analysis was considered a reasonable estimate [6,23,36]. Lastly, some cases have unknown stages in the database and cannot be used in the estimation of screening effect on cancer stages. We calculated the proportion of these cases and found they only account for 3.6% of the total cases. Furthermore, the distribution of participation profiles of all cases changed only slightly after the exclusion of cases with unknown stages, indicating the exclusion of unknown stages has only a minor impact on the participation profiles of the included cases.

### 4.3 Interpretation and policy implications of the findings

In order to achieve the effect of early detection and mortality reduction, the breast cancer screening programme requires more than 70% of eligible women to actually be screened [14]. In our results, the never attenders had the highest risk of advanced stage breast cancer. This group could have benefited from breast cancer screening as regards the reduction of advanced stage cancer, had they participated in screening. Therefore, more intensive effort should be made to encourage the never attenders to participate in screening.

Among women who participate in screening, the irregular attenders have a 17% higher risk of having advanced stage breast cancer than the regular attenders. To achieve regular screening, women do not only have to participate in an adequate number of screenings but also need to participate within the recommended interval. This interval is set at 24 months in Flanders, as it is in many European countries [7]. The benefits and the importance of screening regularity should be highlighted in the breast cancer screening programme promotional materials. Such as the invitation letters and the brochures.

Interestingly, compared with the regular attenders, women who only participated once in screening had a more than two times higher risk of advanced stage breast cancer at diagnosis. This clearly suggests that women who ever participated in screening are a heterogeneous group, and the broad categorization of women into the ever screened and never screened groups in literature may lead to under-estimation of the effect of regular screening. Women who only participated once in screening before they were diagnosed with breast cancer are highly likely to experience symptoms and attend the screening for confirmation. Since symptoms can occur at any age, these women should be encouraged to participate earlier, before they have symptoms, preferably at the age of 50 when they receive their first invitation for screening.

## 5 Conclusions

Never attenders were nearly six times more likely to be diagnosed with advanced stage breast cancer than regular attenders, which was much higher than the effect size that used ever screened women as the reference in literature, indicating that the effect of regular screening was under-estimated in the literature. Irregular screening increases the risk of advanced stage breast cancer by 17%. Women who participate only once in screening are twice as likely to be diagnosed with advanced stage breast cancer, indicating they may have symptoms. The benefit of regular screening, and the risk of not participating in screening until symptoms appear, should be made clear to all women who are eligible for screening.

## Author contributions

Conceptualization: L. Ding, M.J.W. Greuter, G.H. de Bock, G. Van Hal; Data curation: I. Truyen; Formal analysis: L. Ding, I. Truyen; Funding acquisition: G.H. de Bock, G. Van Hal, L. Ding; Investigation: I. Truyen, H. De Schutter, M. Goossens; Methodology: L. Ding, M.J.W. Greuter, G.H. de Bock, G. Van Hal; Project administration: H. De Schutter; Resources: I. Truyen, H. De Schutter, M. Goossens; Software: L. Ding, I. Truyen; Supervision: G.H. de Bock, G. Van Hal; Validation: I. Truyen, H. De Schutter; Visualization: L. Ding, I. Truyen; Writing – original draft: L. Ding, M.J.W. Greuter, G.H. de Bock, G. Van Hal; Writing – review & editing: all authors.

## Data availability statement

The access to the data is possible with the approval from the InterMutualistic Agency, the Belgian Cancer Registry, and the Center for Cancer Detection in Flanders. Further information is available from the corresponding author upon request.

## Ethics statement

Consent from the participants was obtained at the time of screening. Only pseudonymized data were used for this study, and results are reported in an aggregated way. Ethics approval was waived for this study. The study was performed in accordance with the Declaration of Helsinki.

## Funding

The author(s) received no specific funding for this work.

## Declaration of competing interest

The authors declare that they have no known competing financial interests or personal relationships that could have appeared to influence the work reported in this paper.
