# Supplementary material for: Irregular screening participation increases advanced stage breast cancer at diagnosis: A population-based study
Source: Breast. 2022 Jul 8;65:61–6. doi: 10.1016/j.breast.2022.07.004 (PMC9284440; doi:10.1016/j.breast.2022.07.004)
Supplement: Multimedia component 1 [file mmc1.docx]

Table S1 Excluded cases diagnosed after prevalent screen and after only one failed attendance

| Group | Breast cancer cases | Advanced cases |
| --- | --- | --- |
| Prevalent screens related | 6,291 | 469 (7.5%) |
| After one failed attendance | 301 | 103 (34.2%) |
